# Unpacking a time interval lengthens its perceived temporal distance

**DOI:** 10.3389/fpsyg.2014.01345

**Published:** 2014-11-20

**Authors:** Yang Liu, Shu Li, Yan Sun

**Affiliations:** ^1^Key Laboratory of Behavioral Science, Institute of Psychology, Chinese Academy of SciencesBeijing, China; ^2^College of Humanities and Social Sciences, University of Chinese Academy of SciencesBeijing, China

**Keywords:** temporal distance judgment, unpacking effect, quantity estimation, framing effect, time management

## Abstract

In quantity estimation, people often perceive that the whole is less than the sum of its parts. The current study investigated such an unpacking effect in temporal distance judgment. Our results showed that participants in the unpacked condition judged a given time interval longer than those in the packed condition, even the time interval was kept constant between the two conditions. Furthermore, this unpacking effect persists regardless of the unpacking ways we employed. Results suggest that unpacking a time interval may be a good strategy for lengthening its perceived temporal distance.

## Introduction

“*One who works by the minute will own his time fifty-nine times more than those by the hour*.”

-*Rybakov*

Similar to what Rybakov stated, we often *feel* we have more time when thinking of the same time period in a smaller unit. Is it true that people tend to judge a given time interval longer when it is presented in a detailed, rather than compact, way? The answer is not clear owing to lack of scientific evidence. In fact, previous research has proved that temporal distance perception is not equal to objective time. For example, time perception is highly subjective to plenty of factors with regard both to stimulus, such as the size of time duration (Bueti et al., 2008), state of stimuli (dynamic vs. static) (Kanai et al., 2006), and to observer's states, such as attention (Brown and Boltz, 2002; Buhusi and Meck, 2009), emotion (Droit-Volet and Meck, 2007; Tipples, 2008) or medication (e.g., Meck and Church, 1983) (see reviews by Buhusi and Meck, 2009; Burr et al., 2010; Grondin, 2010). Thus, it seems possible that subjective temporal distance judgment could change as a function of the description way of time. The present research adds to the field of temporal distance judgment by examining whether presenting a time interval in a detailed way rather than compact way could lengthen time perception.

A large body of research has already reported that the whole is less than the sum of its parts in quantity estimation (Tversky and Koehler, 1994; Ayton, 1997; Rottenstreich and Tversky, 1997; Fox and Tversky, 1998; Idson et al., 2001; Van Boven and Epley, 2003; Kruger and Evans, 2004; Savitsky et al., 2005; Levine, 2007; Bernasconi et al., 2009; Tsai and Zhao, 2011; Moher, 2012; Haselhuhn, 2014). For example, the sum of subjective probabilities for a person dying from “heart disease, cancer, or other natural causes” tends to be judged greater than the subjective probability for the same person dying simply from “natural causes” (Tversky and Koehler, 1994); the estimated compensation for a type of pollution is bigger when that is described as an origin of “asthma, lung cancer, throat cancer, and all other varieties of respiratory diseases” than as an origin of “all varieties of respiratory diseases (Boven and Epley, 2003). Tversky and Koehler (1994) also regarded the issue of unpacking as a basic principle of human judgment. Considering the similarity between probability and time in human decision-making (Prelec and Loewenstein, 1991; Rachlin and Siegel, 1994; Sun and Li, 2010), we speculate that such an unpacking effect may also be detected in temporal distance judgment.

Furthermore, evidence from recent research was consistent with (but did not directly demonstrate) the unpacking effect in the judgment on temporal distance. Zauberman et al. (2010) revealed that a past event is felt as being more distant when the time interval between the present time and that event is punctuated by a greater number of intervening events. Presumably, punctuating manipulation should unpack a time period, thus lengthening the perceived temporal distance.

In the present study, we directly test the unpacking effect in the perception of temporal distance. Considering previous research focuses on the temporal distance judgment of some events (Kruger and Evans, 2004; Tsai and Zhao, 2011; Min and Arkes, 2012; Moher, 2012; Hadjichristidis et al., 2014), we just use pure manipulation on time interval itself regardless of event and other personal related information. Similar to the probability judgment, we predict that people would judge the same temporal distance longer in the unpacked description (e.g., from June 1st, past by July and August, to September 30th) than in the packed description (e.g., from June 1st to September 30th).

## Methods

### Ethics statement

This study was approved by the Ethics Committee of the Institute of Psychology, Chinese Academy of Sciences. Written consent was obtained from all the participants before the experiment according to the established guidelines of the committee.

### Participants

A total of 122 undergraduates (61% female and 39% male; mean age = 19.9 years) participated in the experiment in exchange for extra course credit. Our sample size was determined by following previous research about unpacking effect (e.g., Tversky and Koehler, 1994; Kruger and Evans, 2004).

### Materials and procedure

Five questions on temporal distance perception were presented in the questionnaire on paper, which had two versions (i.e., packed version and unpacked version). The temporal distance asked in the two versions was the same. The two versions differed only in the packing manipulation. Details of the questions are shown in Table 1 (The whole questionnaires are attached in appendix). The experiment was a between-subjects design: participants were randomly assigned to one of the two versions. Then, they were asked to indicate their perceived temporal distance for each question with a vertical bar “|” on a continuous line scale, in which the left and right anchors of the scale were labeled as very short and very long, respectively. In the end, 61 participants responded to the packed version, and the other 61 participants responded to the unpacked version.

**Table 1:**
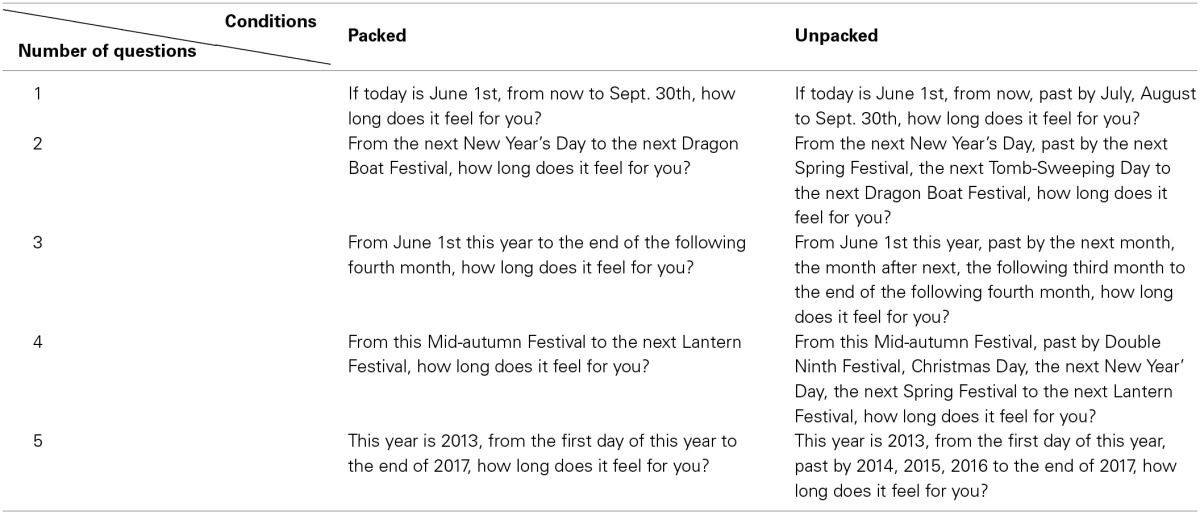
**Questions in packed and unpacked conditions**.

*Note: The Spring Festival, Tomb-Sweeping Day, Dragon Boat Festival, Mid-autumn Festival, Double Ninth Festival, and Lantern Festival are all traditional Chinese festivals*.

We unpacked temporal distance in two different ways, which are usual time expressions in daily life in China, in these five questions. First, we unpacked temporal distance by mentioning natural time unit (e.g., day, month, or year) during the estimated time interval in three questions. Second, we unpacked temporal distance by mentioning holidays (e.g., New Year's Day) during the estimated time interval in the other two questions. The time intervals were equally divided in the unit way, whereas they were not in the holiday way.

## Results and discussion

The distances from the left anchor to the marked point on the scale were recorded for all five questions. Here, longer physical distance suggested longer perceived temporal distance. Thus, the results from an independent samples *t-*test showed that participants in the unpacked condition judged temporal distance longer than those in the packed condition. Among the five questions, the differences were statistically significant in four questions: *t*1 (120) = − 4.311, *p*_1_ < 0.01, *d*_1_ = 0.78; *t*_2_ (120) = −3.976, *p*_2_ < 0.01, *d*_2_ = 0.72; *t*_3_(120) = −2.427, *p*_3_ < 0.05, *d*_3_ = 0.44; *t*_4_ (120) = − 3.690, *p*_4_ < 0.01, *d*_4_ = 0.67, and marginally significant in one question: *t*_5_(120) = − 1.769, *p*_5_ = 0.079, *d*_5_ = 0.32, which is because of the ceiling effect: time horizon in the fifth question is so long. Specific results for each question are listed in Table 2. These results validate our hypothesis that unpacking manipulation can lengthen the perceived temporal distance.

**Table 2:** **The mean distance from the left anchor to the marked point on the scale in each question**.

**Question**	**Condition**	***M* (*SD*) (cm)**	***t***	***P***	***CI***	***d***	**Partial Eta square**
1	Packed	4.94 (2.54)	−4.311	0.000	(−2.97, −1.10)	0.78	0.099
	Unpacked	6.98 (2.67)					
2	Packed	6.40 (2.56)	−3.976	0.000	(−2.82, −0.94)	0.72	0.236
	Unpacked	8.28 (2.67)					
3	Packed	6.22 (2.56)	−2.427	0.017	(−1.92, −0.19)	0.44	0.039
	Unpacked	7.27 (2.24)					
4	Packed	6.22 (2.40)	−3.690	0.000	(−2.40, −0.72)	0.67	0.095
	Unpacked	7.78 (2.25)					
5	Packed	9.28 (2.06)	−1.769	0.079	(−1.30, 0.07)	0.32	0.056
	Unpacked	9.89 (1.75)					

Note that the unpacking effect in the perception of temporal distance was revealed in both the unit-unpacked way (the first, third and fifth questions) and the holiday-unpacked way (the second and fourth questions), which implied that whether the time intervals were equally divided did not affect this effect. In addition, length of time intervals had no influence on the unpacking effect in temporal distance judgment because this effect was detected in time intervals last both short (several months) and long (several years).

## General discussion

In daily life, people often feel that the temporal distance is longer when using small time unit to measure it. To our knowledge, we are the first to report empirical evidence for this feeling. We show that simply splitting one time horizon into many intervals can lengthen perceived temporal distance. Furthermore, we find that this perception is true whether or not the unpacked time interval is equal, and also is true for both the long and short time horizons, thus suggesting the robustness of the unpacking effect in temporal distance judgment.

In the present study, it could be argued that the two time-unpacked ways (unit vs. holiday) may lead to different emotion arousal and episodic memory retrieval since holiday is more special for Chinese. Researchers indeed have demonstrated that these two factors play an important role in temporal distance judgment (e.g., Maricq et al., 1981; Effron et al., 2006; Droit-Volet and Meck, 2007; Broemer et al., 2008; Grondin, 2010). But note that in this study, we focus on the comparison of participants' time perception between the packed and the unpacked descriptions rather than between the unit-unpacked way and the holiday-unpacked way, so we could say that the differences of emotion and memory between date (unit) and holiday did not have influence on our unpacking effect. Moreover, if the unpacking effect discussed here is a result of different emotion arousal or episodic memory retrieval, this effect would disappear or at least attenuate for questions which cause no emotional arousal or episodic memory. But we have even detected the unpacking effect in questions unpacked by mentioning ordinary time unit, which we thought could not lead to emotional arousal or episodic memory. These results indicated that the unpacking effect in temporal distance judgment cannot be explained by emotional arousal and other personal related information (e.g., episodic memory).

Time horizons mentioned in different questions in our study include both past, present and future time. One can ask whether this factor might affect our unpacking effect since prior research (Caruso et al., 2013) revealed that people tend to perceive future events closer than past events of equivalent objective distance. In our experiment, participants in the packed condition and the unpacked condition filled out the questionnaires at the same time, thus there could not be past-future asymmetry of the same question for participants in the packed condition and the unpacked condition and thus this potential confounding variable could be ruled out.

Our unpacking effect in the temporal distance judgment can be explained by the support theory. This theory suggests that the explicit mention of something tends to enhance its salience and hence its support (Tversky and Koehler, 1994). For our cases, given that people do not unpack a time interval naturally, mentioning the relevant time units or holidays included in this time interval might remind and enhance the time salience and thus lengthen its subjective temporal distance judgment. The temporal unpacking effect reported in the present study also helps us understand why unpacking manipulation could work. Tversky and Koehler (1994) came up with two accounts to explain unpacking effects: one is the attentional explanation, which regards unpacking effect as a result of the fact that people are prone to ignore the packed parts of things; the other is the knowledge-based explanation, which means that unpacking effect emerges because people simply have no idea of the subcategories of things. In the present study, since people are all familiar with the unpacked dates or holidays, our results espouse the attentional account rather than the knowledge-based account.

In addition, an alternative account for our findings was the embodied perspective, which insists that metaphors in language not only are used for communication but also represent the internal perceptual information (Barsalou, 2008; Sun et al., 2011; Zhang et al., 2014). Indeed, Nather et al. (2011) indicated that the duration of the posture requiring more movement was perceived longer than the posture requiring less movement. However, so far the connection between the unpacking manipulation and the movement representation has not been demonstrated yet, future work could explicitly examine this connection and figure out whether such a temporal unpacking effect was really mediated by an internal representation of movement.

Generally speaking, time perception plays a significant role in related perceptions, judgments and decisions (Leclerc et al., 1995; Broemer et al., 2008; Wittmann and Paulus, 2008; Kim and Zauberman, 2009). For example, Galak et al. (2014) found that subjective temporal distance judgment from last consumption episode has effect on the next consumption. Thus, our finding here has important implication to a wide range of decision making, such as planning and consumption, in everyday life. Goethe, a famous poet, once said, “We always have time enough, if we will but use it alright.” However, people often bemoan the lack of time in our fast-paced modern society. According to our finding, unpacking a single time interval into several parts may be a good strategy to lengthen its perceived temporal distance, thus offering us a better way to maximize our time.

## Funding

This research was partially supported by National Basic Research Program of China (973 Program, No. 2011CB711000), the National Natural Science Foundation of China (No. 71001098; 31170976).

### Conflict of interest statement

The authors declare that the research was conducted in the absence of any commercial or financial relationships that could be construed as a potential conflict of interest.
